# Sibling psychopathology as a mediator between disability in youth with chronic physical illness and sibling Health-Related quality of life

**DOI:** 10.1186/s12955-025-02471-y

**Published:** 2026-01-03

**Authors:** Dominique Basque, Jennifer Yessis, Scott T. Leatherdale, Mark A. Ferro

**Affiliations:** School of Public Health Sciences, University of Waterloo, 200 University Avenue West, Waterloo, Ontario N2L 3G1 Canada

**Keywords:** Health-related quality of life, Siblings, Chronic disease, Child and youth, Mental disorder, Disability

## Abstract

**Background:**

Chronic physical illnesses (CPIs) can cause significant functional limitations, impacting not only affected youth, but also their siblings. Siblings of youth with chronic physical illness (YwCPI) may experience higher levels psychopathology and poorer health-related quality of life (HRQL). The aim of this study was to assess the mediating role of sibling psychopathology on the association between YwCPI disability and sibling HRQL.

**Method:**

Data come from 171 age-matched siblings (mean age: 9.1years, SD = 4.7 years, 44.4% male) of YwCPI (mean age: 9.5 years, SD = 4.2 years, 50.9% male) participating in a longitudinal study. Parents reported on YwCPI disability (WHODAS 2.0), sibling psychopathology (Emotional Behavioural Scales) and sibling HRQL (KIDSCREEN-27) across five time points over 48 months. Linear mixed-effects models were computed to assess mediation, adjusting for sibling age, sex, and time since CPI diagnosis.

**Results:**

YwCPI disability predicted increased sibling psychopathology (β = 0.31, *p* < 0.001), which in turn was associated with lower sibling HRQL. Indirect effects, indicative of mediation, were significant for all HRQL dimensions (β = -0.19 to -0.07, *p* < 0.05).

**Conclusion:**

Findings demonstrated that sibling psychopathology mediates the impact of YwCPI disability on sibling HRQL, highlighting the importance of identifying and targeting mental health difficulties to promote sibling well-being. Findings reinforce the need for integrated family-centred care approaches to pediatric health services and suggest that family-based supports may mitigate adverse psychosocial outcomes in siblings of YwCPI.

## Introduction

Disability is a holistic term that includes the impairment resulting in the interaction between a health condition and other factors such as life activities, self-care, and peer relationships [1]. It can be used as a proxy-measure for illness severity as it is often a better indicator of poor health outcomes than illness type in youth with chronic physical illness (YwCPI) [2, 3]. Chronic physical illnesses (CPIs), which impact 20% to 45% of youths [4–8], are life-altering conditions that cause functional impairment requiring ongoing treatment to support daily activities. Managing a pediatric CPI extends beyond affected youths and requires families to adapt to health-related challenges, putting other family members at risk of impaired psychosocial functioning [9, 10]. The psychosocial consequences of CPI on siblings, such as worsened peer support [11] and school experiences [12], has garnered attention in youth health research and is supported by family systems theory. This theoretical model underscores changing dynamics of families as they adjust to life events. It posits that emotional functioning is influenced by interactions between an individual and other family members [13]. The external support perceived by siblings of YwCPI from other family members can lead to positive adaptation to CPI and increase cohesion within the family unit [14]. However, lack of support can result in distancing between family members and poor adjustment [14].

Health-related quality of life (HRQL) is a multidimensional concept encompassing the way an individual perceives their health state within the context of their expectations, standards, and interests [15]. The presence of CPI in the family system can directly impact siblings’ psychosocial functioning as their contributions to supporting the chronically ill sibling could place them at risk of having worse HRQL [16–18]. Siblings of YwCPI are more likely to have worse physical well-being [11], school experiences [12], peer support [11], and parent relationships [19], all of which are domains of HRQL. Predictors of HRQL among siblings of YwCPI include illness severity, time since CPI diagnosis, sibling age, sex, and coping skills [17]. Compared to most published reports suggesting sibling HRQL is lower compared to population norms [10, 18, 20], two studies find that siblings of YwCPI have similar HRQL to population norms; likely a function of relatively low illness severity, good illness management, and cultural differences where siblings do not take on caregiving responsibilities [21, 22].

Growing evidence suggests siblings of YwCPI are at elevated risk for psychopathology as they navigate varied emotions including worry, fear, neglect, and jealousy [17, 20, 23–25]. These effects may be exacerbated when YwCPI experience significant disability, as families may prioritize caregiving demands over typical sibling interactions or developmental needs. Research finds many siblings of YwCPI meet clinical cut-offs or borderline clinical symptoms for anxiety, depression, and other mental health problems [20, 26], influenced by factors such as illness severity, and familial and social support [26]. Qureshi et al. (2022) found siblings of YwCPI have similar mental health compared to norms; however, when YwCPI have a co-occurring psychopathology, sibling mental health is poorer compared to norms. Siblings of youth with co-occurring CPI and psychopathology have worse mental health outcomes than those of YwCPI alone [27]. However, this effect is attenuated when adjusting for parent and family factors [27], highlighting the interconnectedness of family members and their role in each other’s well-being [13]. Psychopathology and HRQL are related in pediatric populations; studies suggest impairment of HRQL in siblings of YwCPI can be a consequence of the emotional experience of growing up with an ill family member [15]. Symptoms of psychopathology experienced by siblings can negatively affect physical health, school performance, and social relationships [17, 28], critical components of HRQL.

Overall, evidence suggests levels of disability in YwCPI can impact sibling psychopathology and HRQL. However, research examining the mechanisms underlying these associations are sparse. To our knowledge, no studies have quantified the pathways through which YwCPI disability impacts sibling HRQL. In families with YwCPI, sibling HRQL may be impacted by YwCPI disability as a function of change in sibling psychopathology. The present study addresses this gap by assessing the mediating effect of sibling psychopathology on the association between YwCPI disability and sibling HRQL, as these pathways have not been previously tested within a single model. Identifying the extent to which YwCPI disability and sibling psychopathology contribute to sibling HRQL could offer greater precision in understanding the “why” siblings experience poorer psychosocial outcomes. Understanding these pathways can inform targeted interventions that support psychosocial functioning in siblings and inform models of family-centred care that support and promote optimal outcomes for all family members.

## Method

### Sample

Data come from the Multimorbidity in Children and Youth across the Life-course (MY LIFE) study [29, 30]. This ongoing Canadian study includes five waves of data collection (baseline, 6, 12, 24, and 48 months). Two hundred and sixty-three youths aged between 2 and 16 years with a physician-diagnosed CPI and their primary caregiving parent were recruited from outpatient clinics at McMaster Children’s Hospital [30]. A CPI was defined as one that lasts ≥ 12 months and resulted in at least one of the following: functional limitations, dependencies due to their limitations, and the need for ongoing or additional healthcare [30]. Individuals were excluded from MY LIFE if they were diagnosed with multiple CPIs, or if the youth or parent were not sufficiently proficient in English. For this study, analyses were conducted using data from 171 youths who had an age-matched (± 3 years) sibling.

### Procedure

Families who expressed willingness to participate provided written consent to be contacted by the research team [30]. Informed written consent was obtained from all parents and youth aged ≥ 16 years. Written assent was obtained from youth aged 7 to 15 years, and oral assent was obtained for youth aged < 7 years [30]. Interviews were conducted either at the family home or in hospital. Due to public health protocols implemented in response to the COVID-19 pandemic, by March 2020, all questionnaire data were gathered via mail, and interviews were conducted by telephone. All data used for this study are parent-reported.

### Measures

#### Disability

The World Health Organization Disability Assessment Schedule 2.0 (WHODAS 2.0) is a 12-item that includes six domains: cognition (understanding and communication), mobility (moving and getting around), self-care (hygiene, getting dressed, eating, and staying alone), getting along (interacting with others), life activities (school, responsibilities, and leisure) and participation (joining activities and participation in society) to measure overall health-related disability [31]. Items are assessed on a five-point (0 = ‘none’ to 4 = ‘extreme or cannot do’) Likert scale evaluating the degree of difficulty a person experiences in each domain over the past 30 days. Item scores are summed to obtain a total score; whereby higher scores indicate more disability. The WHODAS 2.0 is valid for youths as young as two years of age [32–35], and demonstrated good internal consistency at baseline (α = 0.86).

#### Symptoms of psychopathology

The Ontario Child Health Study Emotional Behavioural Scales (OCHS-EBS) measures symptoms of psychopathology based on the fifth edition of the Diagnostic and Statistical Manual of Mental Disorders (DSM-5) [36]. The scale includes items that measure symptoms of the seven most common psychopathologies (major depressive disorder, generalized anxiety disorder, separation anxiety, social anxiety disorder, attention-deficit disorder, conduct disorder, and oppositional defiant disorder) among youth. Caregivers are asked to evaluate items on a three-point scale (0 = ‘never or not true’, 1 = ‘sometimes or somewhat true’, and 2 = ‘often or very true’) over the last six months. The raw scores of each item are summed to describe frequency of symptoms; whereby, higher scores correspond to greater psychopathology. The OCHS-EBS was validated in parents of YwCPI in the MY LIFE sample [37], and has excellent internal consistency among siblings in this sample (α = 0.96).

#### Health-related quality of life

The KIDSCREEN-27 is a comprehensive measure of HRQL for youth that measures five dimensions: physical well-being, psychological well-being, autonomy and parent relations, social support and peers, and school environment. The parent-reported version of KIDSCREEN-27 asks respondents to complete the assessment reflecting the perspective of their youth during the past week. Each item is scored from 1 (‘poor’) to 5 (‘excellent’), whereby higher scores indicate better HRQL [38]. The summed score of each dimension is used to calculate a T-score, which is scaled with a mean of 50 and a standard deviation of 10. The KIDSCREEN-27 is reliable and valid among youth as young as two years of age [39]. Internal consistency for all dimensions of the KIDSCREEN-27 ranged from α = 0.79 to 0.92 for siblings in the sample.

### Data analysis

A series of linear mixed-effects models were computed to examine the mediating effect of sibling psychopathology on the association between YwCPI disability and sibling HRQL. Separate models were estimated for each of the five KIDSCREEN-27 dimensions. Fixed effects, random effects, and interaction terms were specified based on theoretical relevance and model fit [40, 41]. Models incorporating higher-order terms were used to assess potential non-linear change in HRQL over time, and nonsignificant higher-order terms were removed in favour of model parsimony. All models were adjusted for sibling age and sex, YwCPI time since diagnosis, and their interactions with time as fixed effects. Sibling age and sex were included as covariates as these factors could confound the measures of associations, while time since diagnosis was included to account for variability in the sample. Covariates were limited to these variables to avoid overadjustment. Time was modeled in years to reflect the spacing between waves of data collection, with values of 0, 0.5, 1, 2, and 4 corresponding with baseline, 6-, 12-, 24-months, respectively. All continuous predictors were grand-mean-centred, and random intercepts were included at the participant level to account for between-person variability at baseline. Reported estimates are standardized, and effect sizes and proportions of mediated effects are used to assess the magnitude of the effects. Given that the purpose of this study is to assess average effects, rather than individual trajectory, a random slope was not included in models. This also serves to maintain model parsimony. Confidence intervals (95%) were calculated based on Wald approximations. Data were assumed to be missing at random as independent t-test and chi-squares found no significant associations between baseline demographics variables and missingness at each time point. Table 1 presents missingness at each time point.

**Table 1:** Missingness at each time point (*N* = 171)

	*n* (%)				
	T1	T2	T3	T4	T5
YwCPI disability	0	14 (8.2)	21 (12.3)	35 (20.5)	46 (26.9)
Sibling psychopathology	1 (0.6)	17 (9.9)	21 (12.3)	35 (20.5)	48 (28.1)
Sibling physical well-being	1 (0.6)	18 (10.5)	22 (12.9)	36 (21.1)	48 (28.1)
Sibling psychological well-being	1 (0.6)	19 (11.1)	22 (12.9)	36 (21.1)	49 (28.7)
Sibling autonomy and parent relation	2 (1.2)	21 (12.3)	25 (14.6)	38 (22.2)	49 (28.7)
Sibling peers and social support	1 (0.6)	20 (11.7)	23 (13.5)	38 (22.2)	49 (28.7)
Sibling school environment	1 (0.6)	20 (11.7)	25 (14.6)	40 (23.4)	52 (30.4)

**Fig. 1:**
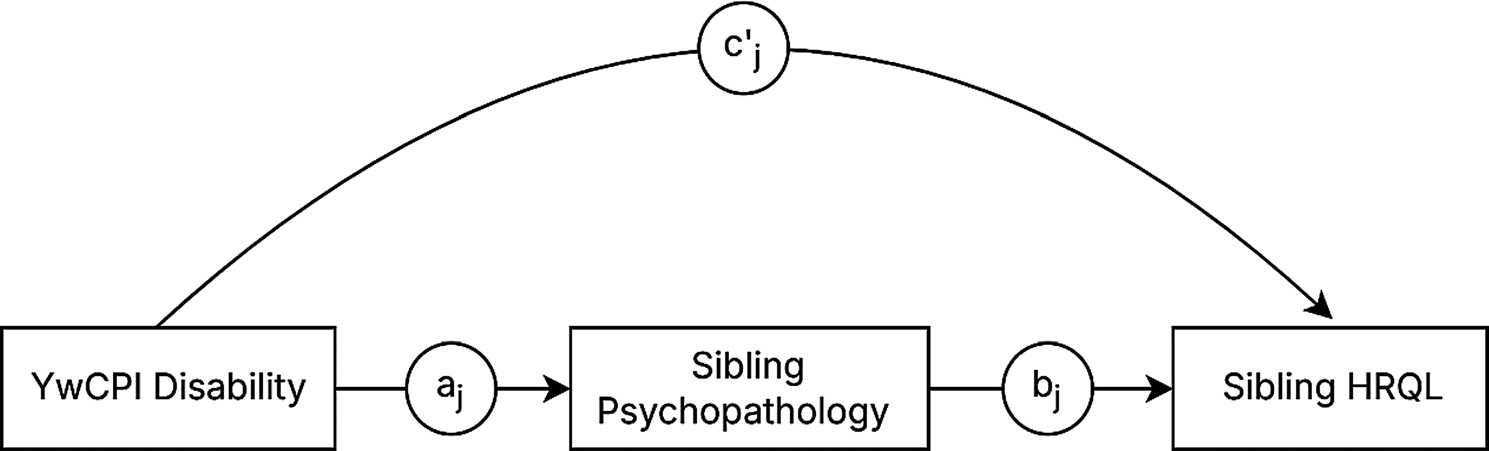
Conceptual diagram of the mediation pathways between disability, sibling psychopathology and sibling HRQ. Note. All models are adjusted for covariates; j denotes the study time points

Mediation analysis followed a standard path model where path *a* represented the effect of YwCPI disability on sibling psychopathology, path *b* reflected the association between sibling psychopathology and sibling HRQL, and path c′ was the direct effect of YwCPI disability on sibling HRQL, adjusting for sibling psychopathology (see Fig. 1). To estimate these paths, two mixed-effect models were constructed. First, a model was specified to estimate path *a*, with sibling psychopathology regressed on YwCPI disability (including covariates). A second model was specified to estimate paths *b* and c′, with sibling HRQL subscales regressed on YwCPI disability and sibling psychopathology, while adjusting for the same covariates. Indirect effects were computed as products of the path *a* and path *b* coefficient [41]. Mediating effects are found when indirect effects are statistically significant. Intra-class correlation coefficients (ICCs) was used to determine the proportion of total variance in HRQL is attributed to between-person differences and within-person fluctuations [42]. An ICC ≥ 0.20 is considered large [43]. Data were analyzed using R version 4.2.1.

## Results

Sociodemographic information is presented in Table 2. Mixed-effects models are shown in Table 3 and mediation path estimates are shown in Table 4. In all five models higher-order terms were nonsignificant and subsequently removed. ICCs ranged between 0.23 and 0.78, suggesting most variance in the outcome was due to between-person differences. Modelling results for the path *a* showed that sibling psychopathology improved over time (β=-0.06, 95% CI[-0.11, -0.00], *p* = 0.049) and greater disability was associated with worse psychopathology (β = 0.14, 95% CI[0.08, 0.21], *p* < 0.001, *d* = 0.167).

**Table 2:** Baseline sociodemographic information of participants in the MY LIFE Study

	**Frequency**	**Percent**
Sibling male	76	44.4
YwCPI male	87	50.9
Physical illness diagnosis		
Rheumatology	52	30.7
Respiratory	39	23.1
Neurological	6	3.6
Haematological	15	8.9
Gastroenterological	22	13.0
Endocrine	18	10.7
Dermatological	18	10.5
**Parent**		
Coupled	155	90.6
Post-secondary graduate	136	79.5
Household income ≥ $90,000	103	60.2
	**Mean**	**SD**
YwCPI age	9.5	4.2
Sibling age	9.1	4.7
Time since diagnosis (years)	4.3	4.1
YwCPI disability	19.7	6.8
Sibling psychopathology	16.4	14.3
Sibling HRQL		
Physical well-being	54.1	12.4
Psychological well-being	52.1	12.0
Autonomy and parent relations	54.9	11.6
Peer and social support	52.6	13.4
School environment	53.0	14.2

**Table 3:** Mixed effect models for mediator and outcomes

Predictors	Estimate ^a^	CI	*p*
**Sibling psychopathology**			
Intercept	-0.01	[-0.21, 0.18]	0.900
YwCPI disability	0.14	[0.08, 0.21]	**< 0.001**
Time	-0.06	[-0.11, -0.00]	**0.049**
YwCPI time since diagnosis	0.02	[-0.13, 0.16]	0.814
Age	0.31	[-0.02, 0.24]	0.103
Female	0.03	[-0.23, 0.28]	0.841
Residual variance ^b^	0.21		
Intercept variance ^c^	0.74		
ICC	0.78		
Conditional R^2^	0.791		
**Physical well-being**			
Intercept	0.13	[-0.03, 0.29]	0.105
YwCPI disability	-0.05	[-0.30, 0.18]	0.244
Time	-0.03	[-1.17, 0.35]	0.307
YwCPI time since diagnosis	0.04	[-0.24, 0.47]	0.522
Age	-0.34	[-1.24, -0.63]	**< 0.001**
Female	-0.23	[-5.78, -0.30]	**0.034**
Psychopathology	-0.29	[-0.42, -0.18]	**< 0.001**
YwCPI disability *time	0.02	[-0.05, 0.08]	0.630
Psychopathology *time	-0.02	[-0.10, 0.06]	0.626
Residual variance ^a^	0.36		
Intercept variance ^b^	0.38		
ICC	0.51		
Conditional R^2^	0.625		
**Psychological well-being**			
Intercept	0.09	[-0.03, 0.22]	0.140
YwCPI disability	-0.03	[-0.10, 0.03]	0.347
Time	-0.05	[-0.10, -0.00]	**0.050**
YwCPI Time since diagnosis	0.07	[-0.02, 0.17]	0.111
Age	-0.26	[-0.35, -0.17]	**< 0.001**
Female	-0.15	[-0.32, 0.01]	0.068
Psychopathology	-0.60	[-0.67, -0.53]	**< 0.001**
Disability *time	0.01	[-0.05, 0.07]	0.776
Psychopathology *time	-0.00	[-0.07, 0.07]	0.975
Residual variance ^b^	0.31		
Intercept variance ^c^	0.21		
ICC	0.40		
Conditional R^2^	0.682		
**Autonomy and parent relations**			
Intercept	0.07	[-0.09, 0.23]	0.410
YwCPI disability	-0.06	[-0.14, 0.02]	0.134
Time	0.01	[-0.05, 0.07]	0.735
YwCPI time since diagnosis	0.03	[-0.08, 0.15]	0.575
Age	-0.11	[-0.23, 0.01]	0.072
Female	-0.11	[-0.32, 0.11]	0.328
Psychopathology	-0.31	[-0.40, -0.22]	**< 0.001**
Disability *time	-0.04	[-0.10, 0.04]	0.351
Psychopathology *time	-0.05	[-0.13, 0.03]	0.236
Residual variance ^b^	0.45		
Intercept variance ^c^	0.37		
ICC	0.45		
Conditional R^2^	0.532		
**Peers and social support**			
Intercept	0.02	[-0.12, 0.17]	0.741
YwCPI disability	-0.04	[-0.12, 0.05]	0.396
Time	-0.12	[-0.20, -0.05]	**0.001**
YwCPI time since diagnosis	0.06	[-0.05, 0.17]	0.281
Age	0.00	[-0.10, 0.11]	0.945
Female	-0.05	[-0.24, 0.14]	0.585
Psychopathology	-0.29	[-0.38, -0.20]	**< 0.001**
YwCPI disability *time	0.01	[-0.07, 0.09]	0.764
Psychopathology *time	-0.06	[-0.15, 0.04]	0.230
Residual variance ^b^	0.71		
Intercept variance ^c^	0.20		
ICC	0.23		
Conditional R^2^	0.300		
**School environment**			
Intercept	-0.06	[-0.21, 0.09]	0.460
YwCPI disability	-0.04	[-0.12, 0.04]	0.309
Time	-0.05	[-0.11, 0.01]	0.11
YwCPI time since diagnosis	0.01	[-0.10, 0.12]	0.829
Age	-0.03	[-0.14, 0.08]	0.584
Female	0.10	[-0.10, 0.30]	0.312
Psychopathology	-0.50	[-0.58, -0.41]	**< 0.001**
YwCPI disability *time	0.04	[-0.02, 0.11]	0.213
Psychopathology *time	-0.02	[-0.10, 0.06]	0.596
Residual variance ^b^	0.42		
Intercept variance ^c^	0.32		
ICC	0.43		
Conditional R^2^	0.576		

*Notes.* ^a^ Estimates are standardized; ^b^ Describes within person variance; ^c^ Describes between person variance

In the physical well-being model, older age (β=-0.34, 95% CI[-1.24, -0.63], *p* < 0.001) and female sex (β=-0.23, 95% CI[-0.43, -0.02], *p* = 0.034) were negatively associated with the outcome (physical well-being). Time (β=-0.05, 95% CI[-0.10, -0.00], *p* < 0.050) and older age (β=-0.25, 95% CI[-0.35, -0.17], *p* < 0.001) were significantly associated with psychological well-being. No covariates were statistically significant in the models for autonomy and parent relations, peer and social support, and school environment.

As for path *b*, elevated symptoms of psychopathology predicted lower physical well-being (β=-0.29, 95% CI[-0.37, -0.21], *p* < 0.001, *d*=-0.478), psychological well-being (β = -0.60, 95% CI[-0.67, -0.53], *p* < 0.001, *d*=-0.986), autonomy and parent relations (β=-0.31, 95% CI[-0.40, -0.22], *p* < 0.001, *d*=-0.508), peer and social support (β=-0.29, 95% CI[-0.38, -0.20], *p* < 0.00, *d*=-0.477), and school environment (β=-0.50, 95% CI[-0.58, -0.41], *p* < 0.001, *d*=-0.809).

**Table 4:** Beta Estimates, 95% confidence intervales, and effect size for paths, indirect effects, and total effects

HRQL Domains	Path a	Path b	Path c’	Indirect effect	Total effect
Physical well-being	**0.14** **[0.81**,** 0.21]** ***d*** **= 0.167**	**-0.29** **[-0.37**,** -0.21]** ***d*** **= -0.478**	-0.44 [-0.12, 0.03] *d* = -0.072	**-0.04** **[-0.06**,** -0.02]** ***d*** **= -0.069**	-0.09 [-0.16, 0.01] *d* = -0.141
Psychological well-being	**0.14** **[0.08**,** 0.21]** ***d*** **= 0.167**	**-0.60** **[-0.67**,** -0.53]** ***d*** **= -0.986**	-0.03 [-0.9, 0.03] *d* = -0.051	**-0.09** **[-0.13**,** -0.05]** ***d*** **= -0.142**	-0.12 [-0.19, 0.04] *d* = -0.192
Autonomy and parent relations	**0.14** **[0.81**,** 0.21]** ***d*** **= 0.167**	**-0.31** **[-0.40**,** -0.22]** ***d*** **= -0.508**	-0.06 [-0.14, 0.02] *d* = -0.099	**-0.05** **[-0.07**,** -0.02]** ***d*** **= -0.073**	**0.11** **[-0.19**,** -0.03]** ***d*** **= -0.172**
Peers and social support	**0.14** **[0.81**,** 0.21]** ***d*** **= 0.167**	**-0.29** **[-0.38**,** -0.20]** ***d*** **= -0.477**	-0.04 [-0.12, 0.05] *d* = -0.06	**-0.04** **[-0.07**,** -0.02]** ***d*** **= -0.069**	0.08 [-0.17, 0.01] *d* = -0.129
School environment	**0.14** **[0.81**,** 0.21]** ***d*** **= 0.167**	**-0.49** **[-0.58**,** -0.41]** ***d*** **= -0.809**	-0.04 [-0.12, 0.04] *d* = -0.065	**-0.07** **[-0.11**,** -0.04]** ***d*** **= -0.116**	**-0.11** **[-0.19**,** -0.03]** ***d*** **= -0.182**

Note. Bold values correspond to the statistically significant values; *d* corresponds to Cohen’s d

In all models, a significant indirect effect between YwCPI disability, through sibling psychopathology, on sibling physical well-being (β=-0.19, CI[-0.28, -0.10], *d*=-0.069), psychological well-being (β =-0.17, 95% CI[-0.23, -0.10], *d*=-0.142), autonomy and parent relations (β=-0.09, 95% CI[-0.14, -0.04], *d*=-0.073), peer and social support (β=-0.07, 95% CI[-0.13, -0.02], *d* =-0.069), and school environment (β=-0.12, 95% CI[-0.19, -0.06], *d*=-0.116) was found, providing evidence of mediation. The proportion of mediated effects were ≥ 42% (physical well-being: 0.49, 95% CI[0.20, 2.21]; psychological well-being: 0.74, 95% CI[0.43, 1.61]; autonomy and parent relations: 0.42, 95% CI[0.19, 1.14], peers and social support: 0.53, 95% CI[-1.73, 3.79]; school environment: 0.64, 95% CI[0.33, 1.91].

## Discussion

This study assessed the mediating effect of sibling psychopathology on the association between YwCPI disability and sibling HRQL. Mediation was found in all five models, suggesting sibling psychopathology could be a target to improve psychosocial outcomes in siblings of YwCPI. Similar to previous findings, greater YwCPI disability was associated with worse sibling psychopathology [20, 26, 44]. This could be attributed to challenges posed by CPI which can lead to negative emotions, and changes to sibling roles and responsibilities [14, 18, 20]. As proposed by family systems theory, such changes could shift family dynamics and impair siblings’ emotional functioning [45]. However, findings in this study suggest siblings adapt to the presence of CPI as their mental health improves over time. The ways in which families adapt to CPI may result in a new normal that buffers the impact of CPI on individual family members. Alternatively, siblings may experience fewer symptoms of psychopathology when they observe YwCPI adapting positively to illness; indeed, findings show two-in-five YwCPI in this sample experience improvements in their mental health over time [46].

### Physical well-being

In siblings, worse psychopathology predicted poorer physical well-being, consistent with previous research [47–50]. However, the direction of this effect remains unclear [47, 50–54]. Given that psychopathology can have somatic manifestations through headaches, fatigue, gastrointestinal issues, and heart palpitations [55], these findings highlight the interconnection between mental and physical health. Other significant predictors were age and sex, where older sisters reported worse physical well-being. Previous studies have reported older sisters are more likely to take on caregiving roles [56–61], which could pose constraints on the time and energy available to engage in physical activity, negatively impacting their physical well-being. Indeed, evidence suggests taking on responsibilities that are not age-appropriate can result in compromised physical health [62]. The interaction between sibling psychopathology and time was nonsignificant, indicating effects of psychopathology on physical well-being remained stable during the study period. This pattern may reflect chronic or persistent effects of psychopathology, rather than evolving or cumulative influences over time. The mediation analysis showed YwCPI disability impacted sibling physical well-being indirectly through increased sibling psychopathology. Therefore, addressing symptoms of psychopathology could be a clinically useful target for improving physical well-being in siblings of YwCPI; evidence shows worse psychopathology contributes to worse physical well-being [63, 64].

### Psychological well-being

Not surprisingly, siblings with higher levels of psychopathology experienced declining psychological well-being over time; those who were older, also reported lower psychological well-being. These findings may reflect the ongoing stress of having a chronically ill sibling, as youth become more aware of the impact of CPI within the family. This could reflect increased knowledge about the illness, prognosis, and medical issues which can result in negative emotions among siblings of YwCPI [65]. Conversely, this finding could be a function of natural development; older age is a consistent predictor of worse psychological outcomes during childhood, adolescence, and young adulthood [66].

The mediation analysis showed that the psychological well-being of siblings of YwCPI may depend on how they experience and cope with CPI, rather than the level of disability. Interventions for siblings focusing on building emotional resilience, and coping mechanisms could mitigate some of the negative psychological consequences associated with YwCPI disability. Evidence from interventions targeting coping skills, particularly problem-focused coping, have shown to improve HRQL [67].

### Autonomy and parent relations

Increased sibling psychopathology was associated with lower scores of autonomy and parent relations in siblings, a finding consistent with previous research [68–70]. In families with a YwCPI, communication between family members tend to have less openness than those without a YwCPI [19], and the quality of parent-child relationships is often a strong predictor of youth psychopathology [14, 26, 71]. Importantly, parent support can play a prominent role in protecting youth mental health [72]. Building on existing literature, these results support the interrelationships of well-being among family members, as outlined by family systems theory [13, 73, 74]. Within this theoretical framework, typical development includes the ability of youths to gain autonomy from the family unit, the absence of which can result in poor emotional well-being [45]. Childhood and adolescence are marked by personal and emotional growth, and development of independence [58]. In siblings of YwCPI, caregiving responsibilities, lower quality of parent-child relationships, and the risk of poor mental health may hinder the development of autonomy. Together, these findings suggest interventions to improve HRQL in siblings should utilize family-centred care approaches that focus on autonomy, communication and strengthening bonds between family members.

### Peers and social support

Sibling psychopathology was significantly associated with lower levels of sibling peer and social support and has been shown to hinder the development and maintenance of supportive peer relationships [72, 75, 76]. Symptoms of psychopathology interfere with social engagement and the quality of peer relationships [77, 78]. However, when parents are unwilling to discuss CPI with siblings they report turning to support groups or the internet for guidance [14]. Encouraging peer support among siblings as a mean of enhancing social functioning could improve overall well-being. Improved psychopathology has been reported in siblings of YwCPI who attend camps, perceive good peer support, and take part in support groups [72].

The indirect effect suggests sibling peer and social relations are shaped by psychopathology and not completely by YwCPI disability. Siblings of YwCPI who experience worse psychopathology may experience additional difficulties in navigating peer relationships and social transition [79], negatively impacting perceived peer and social support. School-based programs, social skills training, and mental health services that may help strengthen peer connections and mitigate the social challenges faced by siblings of YwCPI. Importantly, peer connections can be influenced by many factors. Family environment can influence social skills and opportunities for social engagement [80]. Future research should further examine individual, familial, and social factors that influence peer and social support to disentangle their respective effects.

### School environment

Findings demonstrated a strong negative association between sibling psychopathology and sibling school environment reflecting the extent to which mental health can interfere with academic engagement and adjustment to school demands [81]. Evidence suggests siblings of YwCPI are more likely to exhibit behavioural problems and conflict with peers and teachers, which hinders school performance [17]. Importantly, the perception of peer relationships and teacher-youth interaction, whether positive or negative, influences youth perceptions of school climate [82].

The mediating effect on sibling school environment suggests disability may undermine siblings school experience by exacerbating symptoms of psychopathology, which subsequently compromise engagement and satisfaction in the school setting [83, 84]. Combined with existing evidence, findings show school experience is influenced by both individual psychopathology and broader social interactions in the context of school environment. This confluence underscores the need for multidisciplinary approaches and integrated care models to address psychosocial outcomes among siblings of YwCPI. Addressing psychopathology in schools may foster more supportive and inclusive environments for siblings at risk of disengagement or academic difficulties. This is especially salient during school transition periods, where youth may be vulnerable to declines in psychosocial health [79].

### Implications

This study highlights the role of sibling psychopathology in mediating the impact of YwCPI disability on sibling HRQL. This research is the first to examine and quantify the relationships between YwCPI disability, sibling HRQL, and sibling psychopathology in a single model. Findings suggest sibling psychopathology, rather than disability alone, drives declines in multiple dimensions of sibling HRQL. The results underscore the importance of integrating siblings into family-centred care, with targeted interventions focusing on coping skills, resilience and communication between family members. Routine screening for psychopathology among siblings, in addition to school and peer support, may mitigate negative psychosocial outcomes in siblings of YwCPI.

### Limitations

A few limitations should be considered when interpreting the findings from this study. First, only parent-reported data were used. This could result in common-method bias given that the same parent reported on both siblings, potentially leading to inflated associations. To obtain a more comprehensive understanding of the sibling experience, self-reported and teacher-reported data would provide additional insight into psychosocial outcomes of siblings of YwCPI. Second, the measures used in this study have different recall windows, which could impact the comparability of the measures and introduce measurement variability. Third, no data were available on sibling health (e.g., disability, functioning). Therefore, the impact of sibling health on their HRQL is unknown. Fourth, the relatively small sample size did not allow the use of latent growth curve modelling or random-intercept cross-lagged panel modelling to assess longitudinal mediation, which allows for the simultaneous assessment of direct and indirect effects and testing lagged effects [85].

## Conclusion

This study contributed novel information regarding the critical role of sibling psychopathology in mediating the impact of YwCPI disability on multiple dimensions of sibling HRQL. Together, these findings emphasize the need for screening and intervention targeting sibling psychopathology. Family-centred care and school-based psychological support could alleviate the emotional burden of CPI on siblings, improve coping skills, and enhance psychosocial outcomes. Addressing mental health challenges in siblings is essential for mitigating the broader effects of CPI on family dynamics and individual well-being.

## Data Availability

The data used in this research are not readily available as the authors do not have ethical approval to share the study data. Requests to access the datasets should be directed to Mark A. Ferro, mark.ferro@uwaterloo.ca.

## Abbreviations

YwCPI: Youth with chronic physical illness
CPI: Chronic physical illness
HRQL: Health-related quality of life
MY LIFE: Multimorbidity in Children and Youth across the Life-course
WHODAS 2.0: World Health Organization Disability Assessment Schedule 2.0
OCHS-EBS: Ontario Child Health Study Emotional Behavioural Scales
DSM-5: Diagnostic and Statistical Manual of Mental Disorders
ICC: Intra-class correlation coefficients
